# Chronic Visual Stimulation with LED Light Flickering at 24, 40, or 80 Hz Failed to Reduce Amyloid β Load in the 5XFAD Alzheimer’s Disease Mouse Model

**DOI:** 10.1523/ENEURO.0189-23.2023

**Published:** 2023-08-04

**Authors:** Ya Lan Yang, Ted Weita Lai

**Affiliations:** 1 Graduate Institute of Biomedical Sciences, China Medical University, Taichung 404333, Taiwan; 2 Neuroscience and Brain Disease Center, China Medical University, Taichung 404333, Taiwan; 3 Drug Development Center, China Medical University, Taichung 404333, Taiwan; 4 Translational Medicine Research Center, China Medical University Hospital, Taichung 404327, Taiwan

**Keywords:** 40-Hz flickering light, 5xFAD mouse model, Alzheimer’s disease, amyloid β, γ entrainment, microglia

## Abstract

A single 1-h session (or 7 d of daily 1-h sessions) of noninvasive visual stimulation with LED light flickering at 40 Hz, but not at 20 or 80 Hz, was reported to increase microglial size and decrease amyloid β (Aβ) load in the 5xFAD mouse model of Alzheimer’s disease. To achieve better therapeutic benefits, we explored the effects of daily 1-h sessions of visual stimulation with continuous light or LED light flickering at 24, 40, or 80 Hz for a period of five weeks in 5xFAD mice. As expected, 33-week-old 5xFAD mice but not control wild-type mice of the same age exhibited an abundance of swollen microglia and Aβ plaques in the visual cortex and hippocampus. Unexpectedly, however, compared with similar session of stimulation with continuous light or a light flickering at 24 or 80 Hz, daily sessions of stimulation with LED light flickering at 40 Hz for five weeks failed to further increase the microglial size and could not noticeably decrease the Aβ load in the visual cortex and hippocampus of the 5xFAD mice. In conclusion, contrary to previous findings based on shorter treatment periods, our data showed that daily noninvasive exposure to a light flickering at 40 Hz for a period of five weeks is not effective in reducing Aβ load in the 5xFAD mouse model of Alzheimer’s disease.

## Significance Statement

The recent discovery that a short session of noninvasive visual stimulation with a light flickering at 40 Hz can decrease amyloid β (Aβ) load in a mouse model of Alzheimer’s disease gave hope to the idea that this chronic debilitating disease can be managed by daily sessions of staring at a flickering light. However, we found in this study that daily 1-h sessions involving exposure to a light flickering at 40 Hz for five weeks had no effect on Aβ load in the same mouse model. Our data suggest that visual stimulation alone may not be sufficient for long-term management of Alzheimer’s disease.

## Introduction

Noninvasive visual stimulation with LED light flickering at 40 Hz, but not flickering at 20 or 80 Hz, for 1 h has been reported to entrain γ oscillations and decrease the levels of the amyloid β (Aβ)_1–40_ in the visual cortex in mouse models of Alzheimer’s disease (Iaccarino et al., 2016). Importantly, the decreases in soluble and insoluble Aβ_1–40_ levels and plaque size and number were enhanced when the 1-h visual stimulation sessions were repeated daily for 7 d (Iaccarino et al., 2016). In addition to decreasing Aβ_1–40_ levels, experimental interventions involving daily exposure to LED light flickering at 40 Hz, but not flickering at 80 Hz, for 1 h for a total of 22 d or six weeks have been shown to be neuroprotective in several mouse models of neurodegeneration (Adaikkan et al., 2019). Likewise, repeated twice-daily exposure to a light flickering at 30–50 Hz, but not flickering at 10 or 80 Hz, for 1 h immediately after global ischemia was found to protect the mouse hippocampus against neurodegeneration (Zheng et al., 2020). However, despite these histologic findings in mice, no significant effect of visual stimulation at 40 Hz on clinical outcome in human subjects with Alzheimer’s disease has yet been observed (Ismail et al., 2018; Agger et al., 2023).

Similar to visual stimulation at 40 Hz, auditory stimulus at 40 Hz has been shown to reduce amyloid load in a mouse model of Alzheimer’s disease, and impressively, combined visual and auditory stimulation at 40 Hz was found to provide more widespread benefit than either visual or auditory stimulation alone (Martorell et al., 2019). Importantly, a recent phase 2A clinical study further demonstrated clinical benefits in patients who received three months of combined visual and auditory stimulation at 40 Hz (Chan et al., 2022).

Given that video playbacks from cathode ray tube (CRT) TV displays can entrain the primary visual cortices of macaques and humans (Williams et al., 2004), we were initially interested in exploring whether chronic daily movie watching from a CRT display at certain refresh rates exerts similar beneficial effects in reducing Aβ load as stimulation with LED light flickering at 40 Hz as a positive control (grant #AS-HLGC-110-05). However, we failed to replicate the effect of exposure to LED light flickering at 40 Hz in reducing Aβ load even when mice were treated daily for five weeks. Consistent with our study, another study also failed to reliably demonstrate the benefit of visual stimulation with a light flickering at 40 Hz in mouse models of Alzheimer’s disease (Soula et al., 2023).

## Materials and Methods

### Mice

Male hemizygous 5xFAD (B6SJL-Tg(APPSwFlLon,PSEN1*M146L*L286V)6799Vas/Mmjax, stock #006554, MMRRC #34 840-JAX; Oakley et al., 2006) mice were purchased from The Jackson Laboratory, and control C57BL/6 male mice were purchased from the National Laboratory Animal Center (Taiwan). The animals were cared for in accordance with the ARRIVE (Animal Research: Reporting of In Vivo Experiments) guidelines and the Institutional Guidelines of the China Medical University (CMU) for the Care and Use of Experimental Animals (IGCMU-CUEA), and all experiments were approved by the Institutional Animal Care and Use Committee (IACUC) of CMU (Taichung, Taiwan; protocol No. CMUIACUC-2021-316).

### Visual light stimulation

For each 1-h visual stimulation session, a mouse was placed in a box with bedding; the front-facing wall of the box was transparent to allow light input from a 38-bulb LED light source, and the other three walls were black and opaque to minimize diversion of visual attention from the light source. The ceiling was also transparent to allow for video recording and monitoring of mouse behavior during each visual stimulation session. The light source was preprogrammed by an Arduino single-board microcontroller and generated either continuous light or light flickering at 24, 40, or 80 Hz. The frequency of light flickering was confirmed by manually counting flickers from slow-motion video recordings of selected sessions. The 5xFAD mice subjected to visual light stimulation were 28 weeks of age at the first session of visual stimulation and were euthanized at 33 weeks of age after five weeks of daily 1-h visual stimulation sessions.

### Immunofluorescence

Immunofluorescence staining of microglia and Aβ was performed with primary antibodies against Iba1 (1:500; GeneTex, catalog #GTX100042) and Aβ (1:500; Cell Signaling, catalog #8243), respectively, followed by an anti-rabbit secondary antibody (1:1000; Abcam, catalog #ab150080), as described previously (Wu and Lai, 2021). The area occupied by microglia and Aβ were measured using the image analysis software ImageJ (NIH) by an investigator blinded to the treatment groups.

### Statistical analysis

The data are presented as the mean ± SEM. The area occupied by microglia and Aβ plaques in the visual cortex and hippocampus was compared between control C57BL/6 mice and 5xFAD mice by unpaired *t* test. The effects, or lack thereof, of daily 1-h visual stimulation with continuous light or light flickering at 24, 40, or 80 Hz for five weeks were analyzed by one-way ANOVA.

## Results

### Chronic visual stimulation with light flickering at 40 Hz failed to increase the area occupied by microglia in the visual cortex and hippocampus of 5xFAD mice

**Figure 1.**
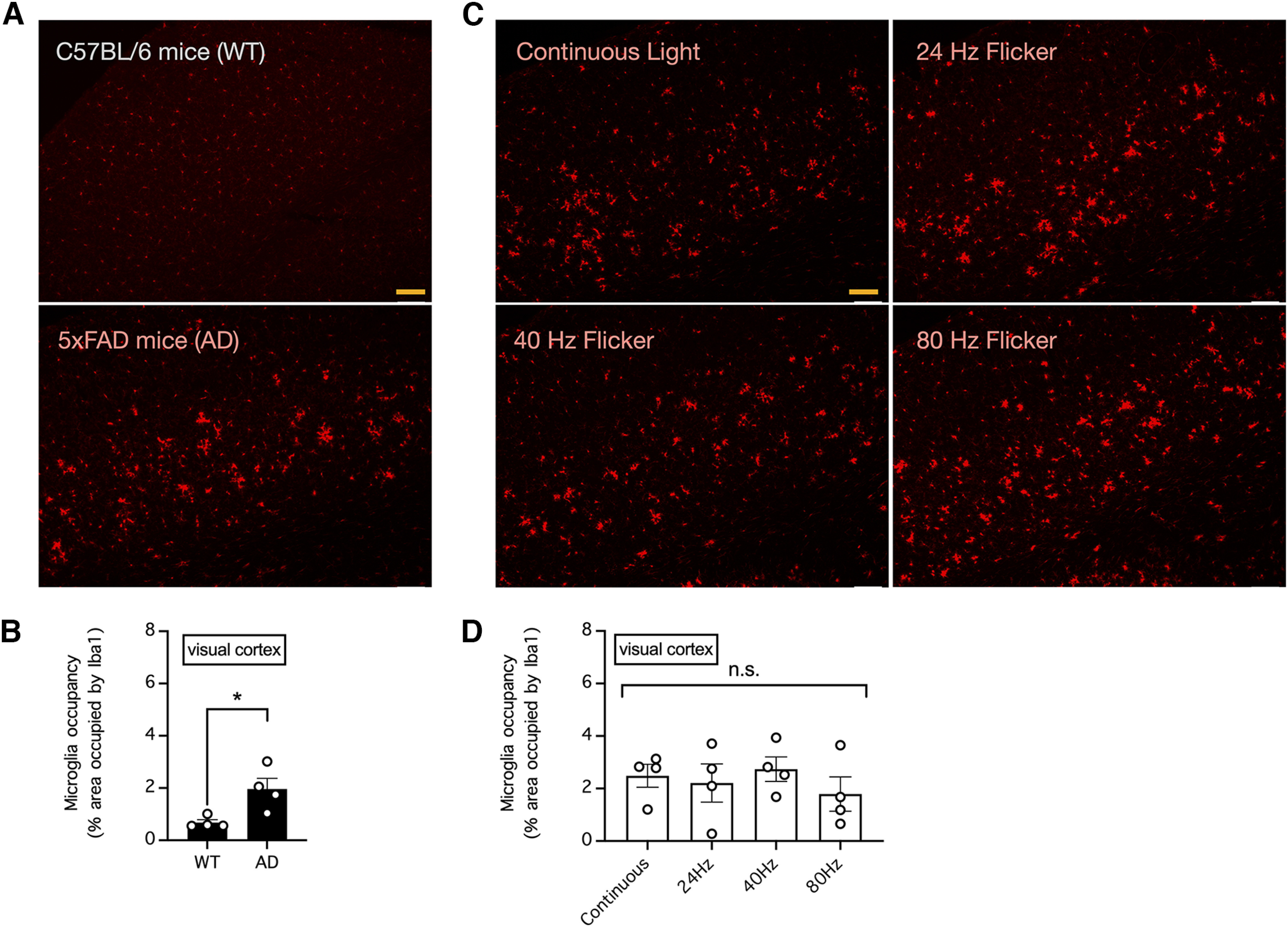
Visual stimulation with light flickering at 40 Hz had no effect the area occupied by microglia in the mouse visual cortex. ***A***, The relative area occupied by microglia in the visual cortex of 33-week-old control C57BL/6 and 5xFAD mice was determined by Iba1 immunostaining. Bar indicates 200 μm. ***B***, Summary of the results in ***A*** showing the relative area occupied by microglia in the visual cortex. *n* = 4 mice per group. * indicates *p *<* *0.05 when compared by unpaired *t* test. ***C***, The relative area occupied by microglia in the visual cortex of 5xFAD mice subjected to daily 1-h visual stimulation with continuous light or light flickering at 24, 40, or 80 Hz for five weeks was determined by Iba1 immunostaining. Bar indicates 200 μm. ***D***, Summary of the results in ***C*** showing the relative area occupied by microglia in the visual cortex. *n* = 4 mice per group. n.s. indicates no significant difference when compared by one-way ANOVA.

**Figure 2.**
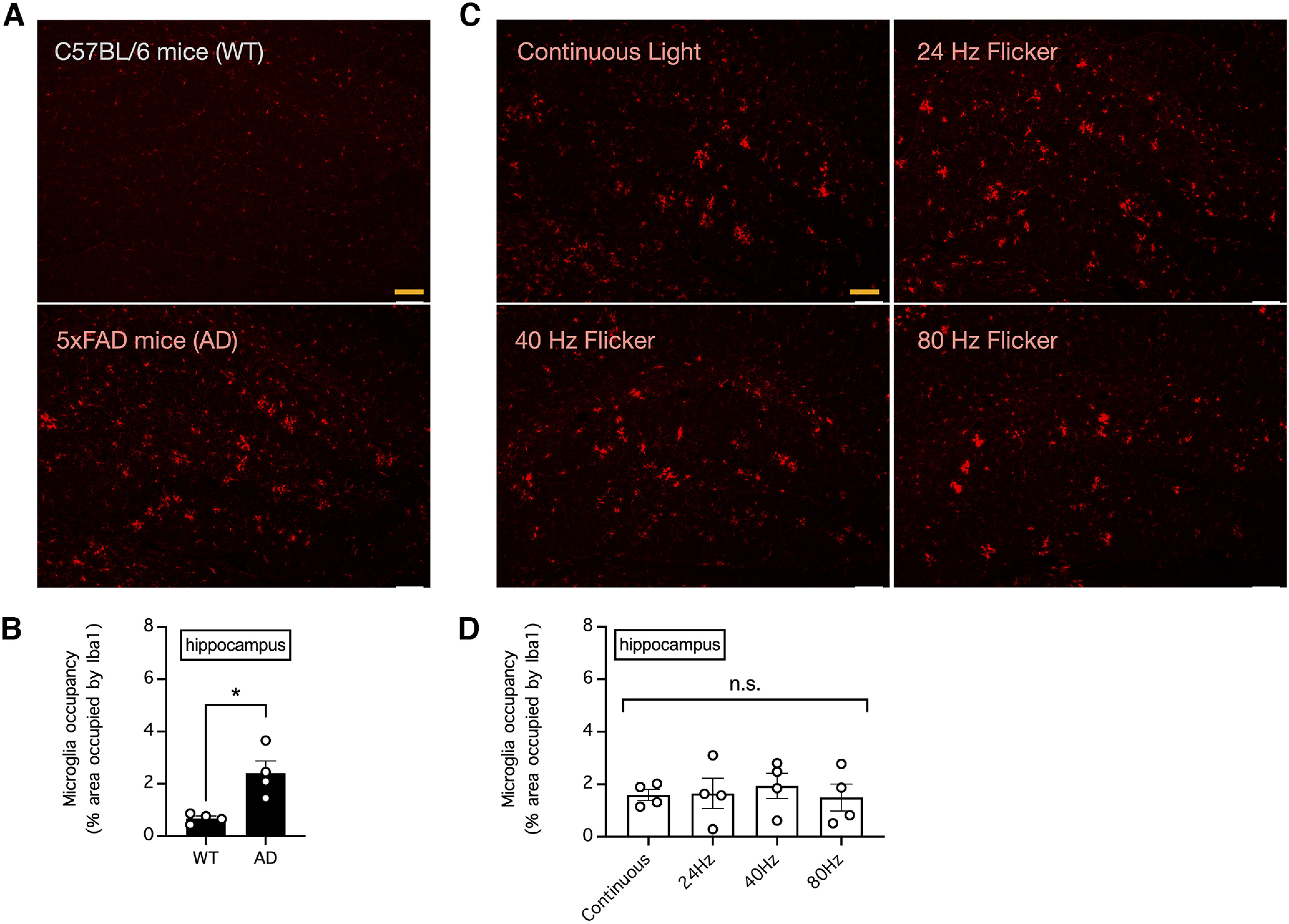
Visual stimulation with light flickering at 40 Hz had no effect on the area occupied by microglia in the mouse hippocampus. ***A***, The relative area occupied by microglia in the hippocampus of 33-week-old control C57BL/6 and 5xFAD mice was determined by Iba1 immunostaining. Bar indicates 200 μm. ***B***, Summary of the results in ***A*** showing the relative area occupied by microglia in the hippocampus. *n* = 4 mice per group. * indicates *p *<* *0.05 when compared by unpaired *t* test. ***C***, The relative area occupied by microglia in the hippocampus of 5xFAD mice subjected to daily 1-h visual stimulation with continuous light or light flickering at 24, 40, or 80 Hz for five weeks was determined by Iba1 immunostaining. Bar indicates 200 μm. ***D***, Summary of the results in ***C*** showing the relative area occupied by microglia in the hippocampus. *n* = 4 mice per group. n.s. indicates no significant difference when compared by one-way ANOVA.

A hallmark of Alzheimer’s disease in mice is neuroinflammation associated with enlarged and activated microglia in the brain; notably, it has been reported that visual stimulation by light flickering at 40 Hz can further increase the microglial cell body diameter, indicating enhanced Aβ engulfment, in the visual cortex of 5xFAD mice (Iaccarino et al., 2016). To explore the abovementioned phenomenon, we compared microglia in the visual cortex and hippocampus between 33-week-old control C57BL/6 mice and 5xFAD mice of the same age (Figs. 1*A*,*B*, 2*A*,*B*). Consistent with the observation of neuroinflammation in the brains of Alzheimer’s disease mice, we found an abundance of large swollen microglia in 5xFAD mice but not in control mice (Figs. 1*A*, 2*A*); therefore, the area occupied by microglia in the visual cortex and hippocampus was significantly larger in 5xFAD mice than in control mice (*n* = 4 mice per group; *p *=* *0.0235 for the difference in the area occupied by microglia in the visual cortex and *p *=* *0.0104 for the difference in the area occupied by microglia in the hippocampus, unpaired *t* tests; Figs. 1*B*, 2*B*). To confirm whether chronic exposure to light flickering at 40 Hz can further increase the microglial cell body diameter, we subjected 5xFAD mice to daily 1-h visual stimulation with continuous light or light flickering at 24, 40, or 80 Hz for five weeks (Figs. 1*C*,*D*, 2*C*,*D*). However, there was no apparent effect of flickering light on the microglia cell body diameter in these mice (Figs. 1*C*, 2*C*), and therefore, there was no significant difference in the area occupied by microglia in the visual cortex or deeper brain structures such as the hippocampus (*n* = 4 mice per group; *p *=* *0.6999 for the difference in the area occupied by microglia in the visual cortex and *p *=* *0.9194 for the difference in the area occupied by microglia in the hippocampus, one-way ANOVA; Figs. 1*D*, 2*D*).

### Chronic visual stimulation with light flickering at 40 Hz failed to decrease the Aβ load in the visual cortex and hippocampus of 5xFAD mice

**Figure 3.**
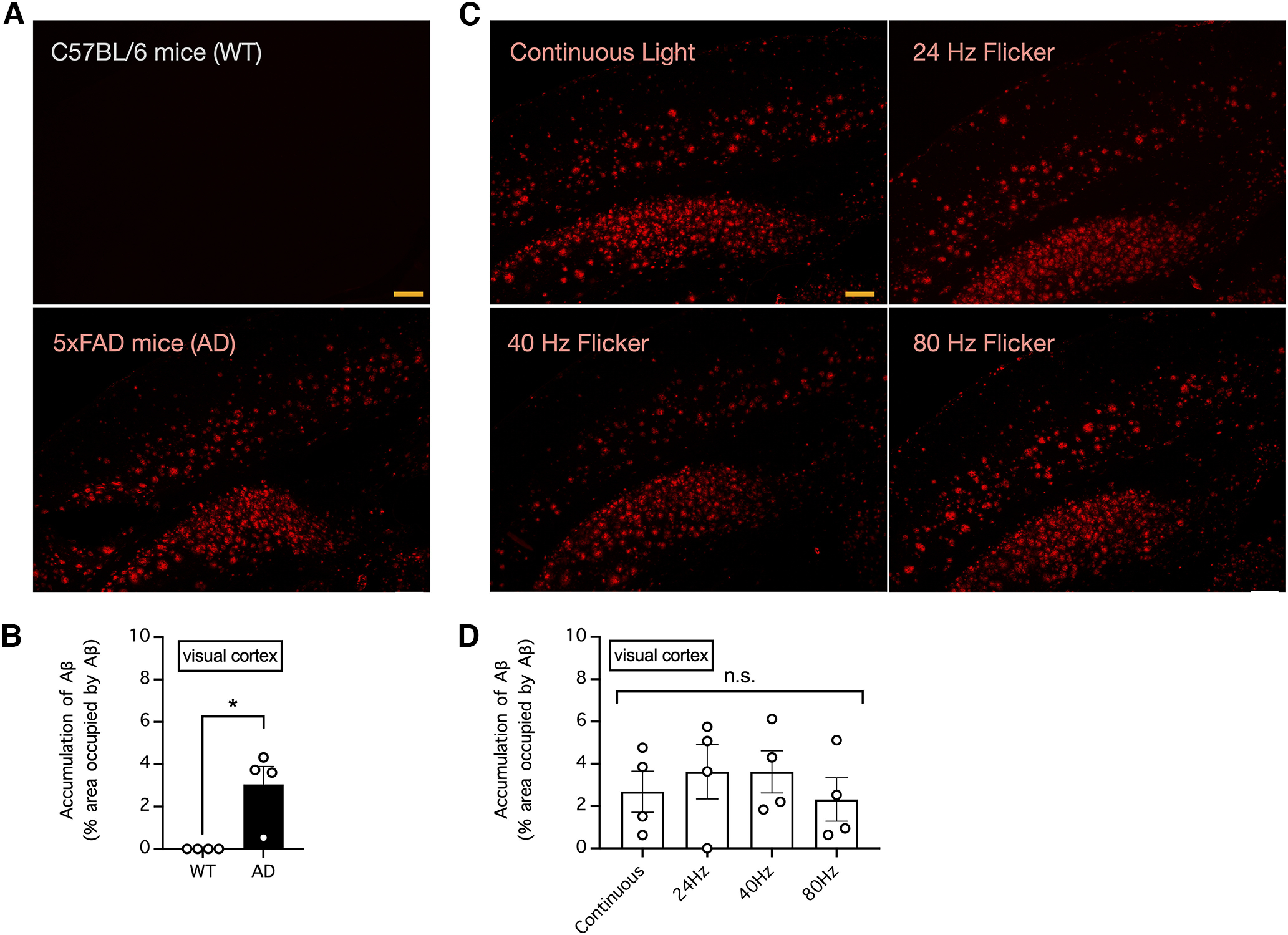
Visual stimulation with light flickering at 40 Hz had no effect on endogenous levels of amyloid β (Aβ) in the mouse visual cortex. ***A***, The endogenous levels of Aβ in the visual cortex of 33-week-old control C57BL/6 and 5xFAD mice were determined by Aβ immunostaining. Bar indicates 200 μm. ***B***, Summary of the results in ***A*** showing the relative area occupied by Aβ in the visual cortex. *n* = 4 mice per group. * indicates *p *<* *0.05 when compared by unpaired *t* test. ***C***, The endogenous levels of Aβ in the visual cortex of 5xFAD mice subjected to daily 1-h visual stimulation with continuous light or light flickering at 24, 40, or 80 Hz for five weeks were determined by Aβ immunostaining. Bar indicates 200 μm. ***D***, Summary of the results in ***C*** showing the relative area occupied by Aβ in the visual cortex. *n* = 4 mice per group. n.s. indicates no significant difference when compared by one-way ANOVA.

**Figure 4.**
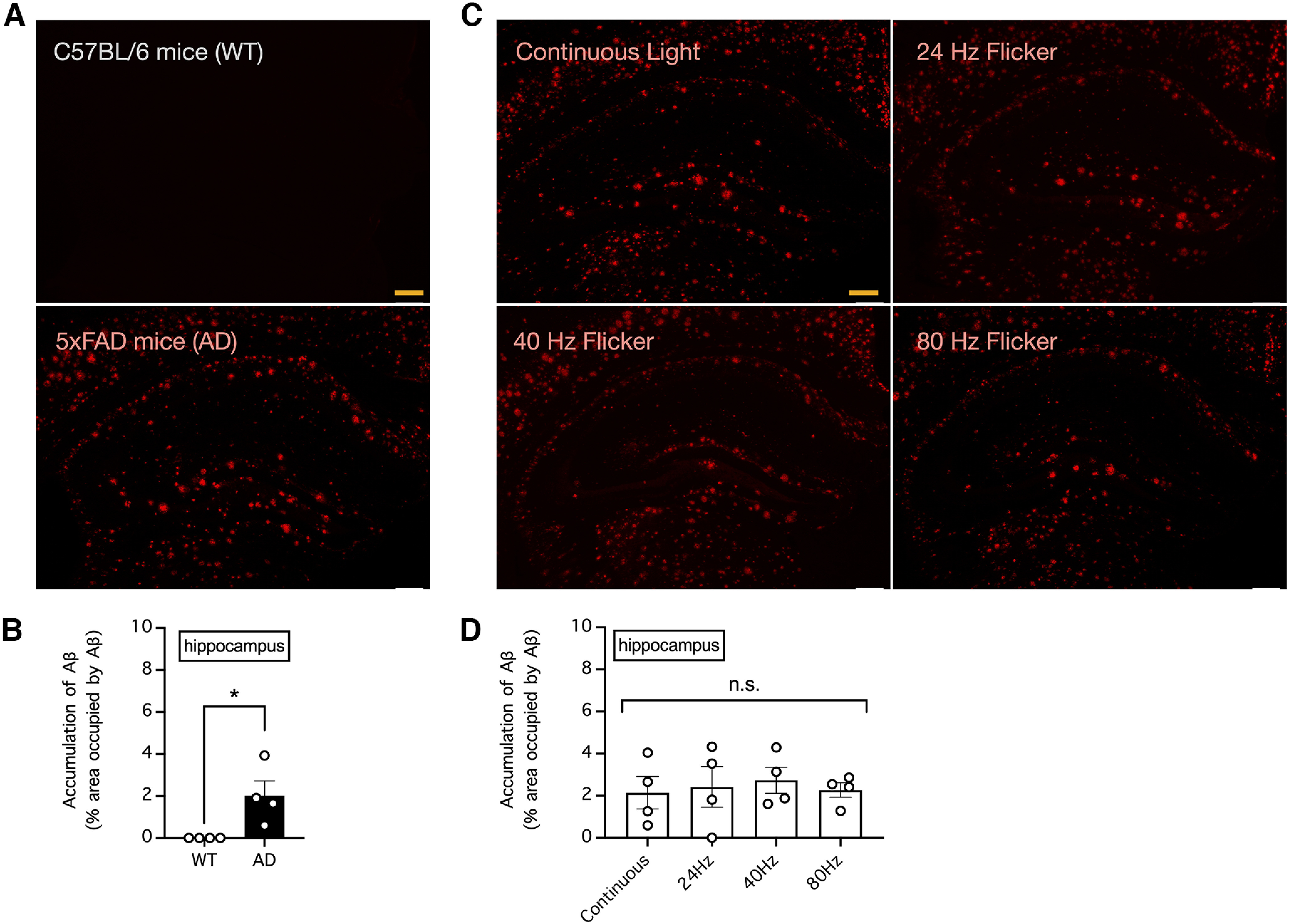
Visual stimulation with light flickering at 40 Hz had no effect on endogenous levels of amyloid β (Aβ) in the mouse hippocampus. ***A***, The endogenous levels of Aβ peptides in the hippocampus of 33-week-old control C57BL/6 and 5xFAD mice were determined by Aβ immunostaining. Bar indicates 200 μm. ***B***, Summary of the results in **A** showing the relative area occupied by Aβ in the hippocampus. *n* = 4 mice per group. * indicates *p *<* *0.05 when compared by unpaired *t* test. ***C***, The endogenous levels of Aβ in the hippocampus of 5xFAD mice subjected to daily 1-h visual stimulation with continuous light or light flickering at 24, 40, or 80 Hz for five weeks were determined by Aβ immunostaining. Bar indicates 200 μm. ***D***, Summary of the results in ***C*** showing the relative area occupied by Aβ in the hippocampus. *n* = 4 mice per group. n.s. indicates no significant difference when compared by one-way ANOVA.

To explore whether chronic exposure to light flickering at 40 Hz can significantly decrease the Aβ load in 5xFAD mice, we immunostained brain slices from the abovementioned mice for Aβ. Consistent with the observation of amyloidosis in mice with Alzheimer’s disease but not in control mice, we found a large amount of Aβ in the visual cortex and hippocampus of 5xFAD mice at 33 weeks of age but not in control C57BL/6 mice of the same age (Figs. 3*A*,*B*, 4*A*,*B*). As a result, the relative area of the visual cortex and hippocampus occupied by Aβ was significantly higher in 5xFAD mice than in control mice (*n* = 4 mice per group; *p *=* *0.0118 for the difference in the area occupied by Aβ in the visual cortex and *p *=* *0.0273 for the difference in the area occupied by Aβ in the hippocampus, unpaired *t* tests; Figs. 3*B*, 4*B*). However, in 5xFAD mice subjected to daily 1-h visual stimulation with continuous light or light flickering at 24, 40, or 80 Hz for five weeks, we found no apparent effect of light flickering in decreasing Aβ load (Figs. 3*C*, 4*C*) and thus no significant difference in the area occupied by Aβ in the visual cortex or deeper brain structures such as the hippocampus (*n* = 4 mice per group; *p *=* *0.7682 for the difference in the area occupied by Aβ in the visual cortex and *p *=* *0.9415 for the difference in the area occupied by Aβ in the hippocampus, one-way ANOVA; Figs. 3*D*, 4*D*).

## Discussion

A single session of 1-h noninvasive visual stimulation with light flickering at 40 Hz was shown to be sufficient to increase the average microglia size and decrease the Aβ load in 5xFAD mice, while similar sessions of stimulation with continuous light or light flickering at 20 or 80 Hz was found to have no effect on the Aβ load (Iaccarino et al., 2016). Therefore, we anticipated that five weeks of daily 1-h visual stimulation with light flickering at 40 Hz would be even more effective than a single session in increasing microglial size and decreasing the Aβ load in 5xFAD mice, whereas similar treatment with continuous light or light flickering at 20 or 80 Hz would have no effect. Unexpectedly, however, we found no difference in microglial size or Aβ load in 5xFAD mice chronically treated with either continuous light or light flickering at 24, 40, or 80 Hz. One possible explanation for the difference between the effects of chronic visual stimulation in our study and the effects of a single session of visual stimulation reported previously (Iaccarino et al., 2016) is tolerance to the therapeutic effects of light flickering at 40 Hz on Aβ load after repeated daily treatments. Notably, however, this explanation contradicts other recent studies showing that chronic daily 1 h or twice-daily visual stimulation with light flickering at 30–50 Hz, but not similar treatment with light flickering at 80 Hz, for up to six weeks is neuroprotective in many mouse models of neurodegenerative diseases (Adaikkan et al., 2019; Zheng et al., 2020). Interestingly, consistent with our study, another research group reported no significant change in microglial size and Aβ plaque load in 5xFAD mice subjected to either a single 1-h session of visual stimulation with light flickering at 40 Hz or 7 d of daily 1-h sessions of visual stimulation with light flickering at 40 Hz (Soula et al., 2023). Additionally, it is worth noting that these negative results for stimulation with light flickering at 40 Hz in 5xFAD mice are consistent with data from early clinical trials in which human subjects with Alzheimer’s disease showed no clinical benefit from visual stimulation with light flickering at 40 Hz (Ismail et al., 2018; Agger et al., 2023).

The explanation for why the effect of light flickering at 40 Hz on microglia and Aβ load in 5xFAD mice could not be replicated by our laboratory and another laboratory may be multifactorial (see Table 1, which compares the experimental protocols and animals used between this and other studies). For instance, to minimize diversion of attention of the treated mouse from the light source, we made all of the walls of the treatment box opaque except for the transparent wall facing the light source to limit other visual input from the surroundings. However, we cannot ensure that the mice were not closing their eyes or looking at the floor or opaque walls. Indeed, it has been shown that when given the choice, mice would prefer to spend time in a compartment with continuous light rather than a compartment with light flickering at 40 Hz (Soula et al., 2023). In addition, the very large variability in microglial morphology and Aβ load between individual 5xFAD mice in this and similar studies could also obscure the effects of light flickering at 40 Hz, particularly if the effects are modest in magnitude.

**Table 1:** Comparison of experimental protocols, animals used, and findings between previous studies and the present study

	Iaccarino et al. (2016), Nature 540:230–235			Soula et al. (2023), Nature Neuroscience 26:570–578			Present study
Alzheimer’s disease model	5xFAD mice		TauP301S mice	5xFAD mice		5xFAD and APP/PS1 mice	5xFAD mice
Animal gender	Male		Male	Male and female		Not specified	Male
Animal age	3 months old	6 months old	4 months old	4 months old	7 months old	7 months old	1
Visual stimulation protocol	LED light flickering at 40 Hz for 1 h	LED light flickering at 40 Hz for 1 h	LED light flickering at 40 Hz for 1 h	LED light flickering at 40 Hz for 1 h	LED light flickering at 40 Hz for 1 h	LED light flickering at 40 Hz for 1 h	LED light flickering at 40 Hz for 1 h
Number of sessions	A single session	Repeat daily for 7 d	Repeat daily for 7 d	A single session	Repeat daily for 7 d	A single session	Repeated daily for 5 weeks
Effect on microglial cell body diameter and the area occupied by microglia	Increase in microglial cell body diameter and the area occupied by microglia in the visual cortex	Not determined	Increase in microglial cell body diameter and the area occupied by microglia in the visual cortex	Not determined	Not determined	No difference in microglial cell body diameter and the area occupied by microglia in the visual cortex	No difference in the area occupied by microglia in the visual cortex and hippocampus
Effect on Aβ plaque load	Not determined	Decreased Aβ plaque number and size by >60% in the visual cortex	Not determined	No difference in the area occupied by Aβ load in the visual cortex and hippocampus	No difference in the area occupied by Aβ load in the visual cortex and hippocampus	No difference in the area occupied by Aβ load in the visual cortex	No difference in the area occupied by Aβ load in the visual cortex and hippocampus
Number of animals tested	*n* = 4 mice per group × 2 groups	*n* = 8 mice per group × 2 groups	*n* = 8 mice per group × 2 groups	*n* = 20–22 mice per group × 2 groups	*n* = 11–12 mice per group × 2 groups	*n* = 3–7 mice per group × 2 groups	*n* = 4 mice per group × 4 groups
Statistical analysis used	Unpaired *t* test	Unpaired *t* test	Unpaired *t* test	Two-sided Wilcoxon test	Two-sided Wilcoxon test	Two-sided Wilcoxon test	One-way ANOVA

^1^Seven-month-old at the first visual stimulation session, and eight-month-old after the last session.

In conclusion, although we cannot rule out the possibility that the failure to replicate the effects of flickering light in 5xFAD mice could be because of the very large variability in Aβ load between individual 5xFAD mice, lack of visual attention to and focus on the light source during treatment sessions, or both, our study highlights the need to further explore this subject and question whether the lack of changes in Aβ load in mice could explain the lack of clinical benefit in early clinical trials. Moreover, in comparison to visual stimulation alone, combined visual and auditory stimulation at 40 Hz have shown greater therapeutic efficacy in mouse models and promising results in a recent phase 2A clinical trial (Martorell et al., 2019; Chan et al., 2022). Therefore, it is possible that the lack of experimental and clinical benefit of visual stimulation alone in some studies is because the effects are too subtle to be reliably observed and quantified.

## Synthesis

Reviewing Editor: Karl Herrup, University of Pittsburgh School of Medicine Decisions are customarily a result of the Reviewing Editor and the peer reviewers coming together and discussing their recommendations until a consensus is reached. When revisions are invited, a fact-based synthesis statement explaining their decision and outlining what is needed to prepare a revision will be listed below. The following reviewer(s) agreed to reveal their identity: Zoltan Simandi.

This study is a failure-to-replicate report on the oft-cited Nature paper by Iaccarino et al on the benefit of 40 Hz flickering light as a therapy for Alzheimer’s disease. The original paper applied one-hour sessions (either single or repeated) of stimulation with flickering LED light at different frequencies. In the 5xFAD mouse model they found an increase microglial size and a decrease Aβ burden. The authors of the current study used daily 1-h sessions of stimulation with continuous light or flickering LED light for 5 weeks in the same mouse model. They validated the microglial and amyloid phenotypes untreated animals, but were unable to demonstrate either an increase microglial size or a notable decrease Aβ load. Though the original findings were based on shorter treatment periods, the authors show that a nearly identical light therapy for 5 weeks had little or no effect. The findings are of considerable importance to the Alzheimer’s disease field as human trials are already underway based on the Iaccarino study.

There was agreement that the study provides compelling data that chronic visual stimulation with LED light in 5x FAD mouse model of AD did not decrease the Aβ load in the visual cortex and hippocampus and that overall, this is an important cautionary study. The findings are basically well done, but important changes are needed to strengthen the paper.

A general comment is that in cases such as this where the authors are describing what are essentially negative results, it is imperative to use the exact methods employed in the previous studies. It is unclear, for example, whether the authors are suggesting that LED light is having no effect or instead that short stimulation (as performed by Iaccarino) works but chronic stimulation (as in the current study) does not. It would assist the reader if a table were provided that includes the previous experimental setups (both Iaccarino et al., 2016 and the more recent Soula et al., 2023) side by side with the current findings. The table should list the various studies/parameters tested and whether or not there was an effect. Experimental design features such as the specific AD model used, the number of animals tested, the age of the animals, their gender, etc. should all be included.

There were minor proof-reading errors as well. At one point the authors cite support as coming from “grant #XXX”. At another, the report that “The Jackson Laboratory (USA), and control C57BL/6 male mice were purchased from the XXX (XXX). The animals were cared for in accordance with the ARRIVE guidelines and the Institutional Guidelines of XXX (XXX) for the Care and Use of Experimental Animals (XXX), and all experiments were approved by the Animal Care Committee of XXX (XXX, XXX) (protocol No. XXX).” These errors must be corrected.

The Methods section needs to include language that lets the reader know whether or not the investigators were blinded to the treatment groups when conducting the analysis.
